# Bullous pemphigoid: a rare clinical image

**DOI:** 10.11604/pamj.2024.47.203.43265

**Published:** 2024-04-22

**Authors:** Shraddha Patil, Archana Maurya

**Affiliations:** 1 Department of Child Health Nursing, Smt. Radhikabai Meghe Memorial College of Nursing Datta Meghe Institute of Higher Education and Research (Deemed University) Sawangi Wardha, Maharashtra, India

**Keywords:** Bullous pemphigoid, elderly, autoimmune disorder, blistering disease, corticosteroids

## Image in medicine

Bullous pemphigoid (BP) is the most common autoimmune blistering illness, accounting for 70% to 80% of all subepidermal immunobullous cases. Bullous pemphigoid primarily affects the elderly, especially those who are between 60 to 80 years of age. Bullous pemphigoid is a rare autoimmune blistering illness characterized by the development of autoantibodies directed towards basement membrane zone components such as BP180 and BP230. It primarily affects the elderly, with a peak occurrence during the seventh and eighth decades of life. We present a rare case of an 80-year-old male with a bullous pemphigoid. The patient reported widespread blistering and erosions on his back skin, and upper and lower arms, as well as itching, discomfort, and pain. The patient was presented to the hospital with a serious complaint of tense blisters. Blisters are frequently seen on the arms, and upper and lower extremities, the patient is taking corticosteroids, prednisolone, intravenous immunoglobin, and anti-inflammatory drugs. A skin biopsy is recommended for the patient. There are various therapeutic options for this sickness, including anti-inflammatory medications, pharmaceuticals that decrease antibody formation, and treatments that improve antibody removal. Systemic corticosteroids are frequently used as first-line therapy, sometimes in conjunction with immunosuppressive medications like azathioprine or mycophenolate mofetil in resistant cases. Emerging drugs, such as rituximab and intravenous immunoglobulin, show promise in situations where standard therapy fails. Despite therapeutic improvements, blood pressure remains a complex illness linked with significant morbidity and death, emphasizing the need for ongoing research to enhance diagnostic procedures and create tailored therapeutics.

**Figure 1:**
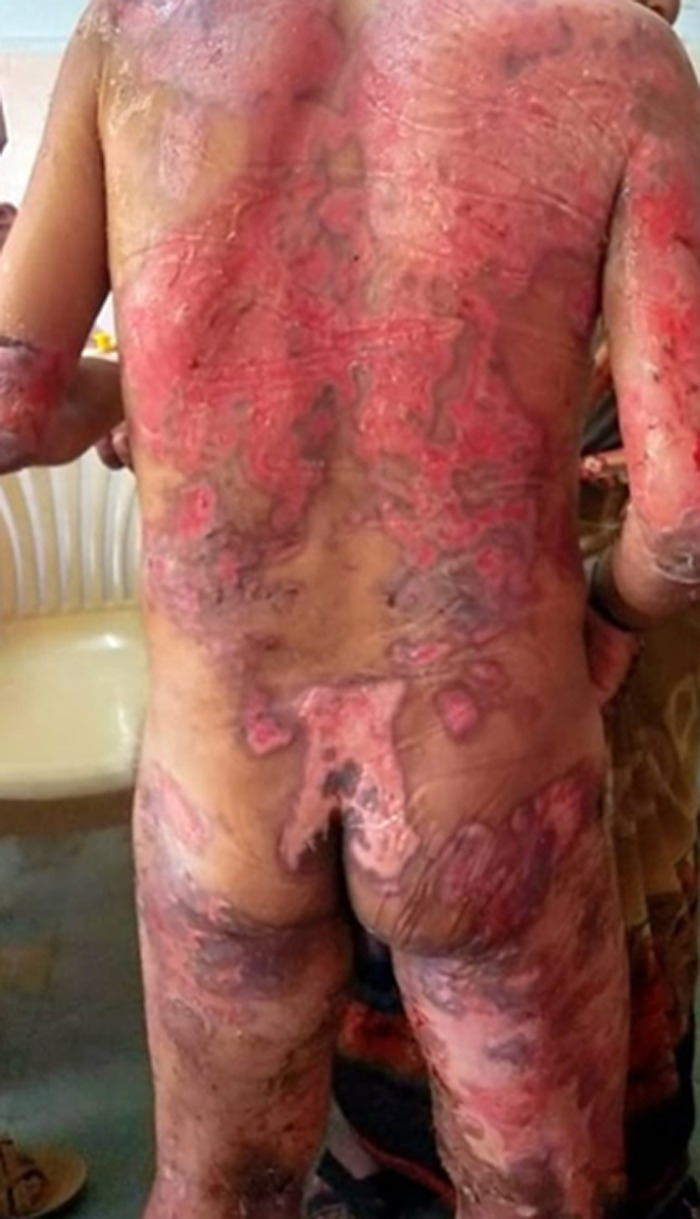
widespread blistering and erosions over his back skin, upper and lower arm

